# Safety and antibody response to two-dose SARS-CoV-2 messenger RNA vaccination in patients with multiple myeloma

**DOI:** 10.1186/s12885-021-09097-5

**Published:** 2021-12-27

**Authors:** Ross S. Greenberg, Jake A. Ruddy, Brian J. Boyarsky, William A. Werbel, Jacqueline M. Garonzik-Wang, Dorry L. Segev, Philip H. Imus

**Affiliations:** 1 Department of Surgery, Johns Hopkins University School of Medicine, Baltimore, MD USA; 2 Department of Infectious Diseases, Johns Hopkins University School of Medicine, Baltimore, MD USA; 3 Department of Epidemiology, Johns Hopkins University Bloomberg School of Public Health, Baltimore, MD USA; 4 Department of Oncology, Johns Hopkins University School of Medicine, Baltimore, MD USA

**Keywords:** Multiple myeloma, COVID-19, SARS-CoV-2, mRNA vaccination, Antibody

## Abstract

**Background:**

Patients with multiple myeloma (MM) were excluded from the original SARS-CoV-2 mRNA vaccine trials, which may influence vaccine hesitancy in this population. We prospectively characterized the safety and immunogenicity of two-dose SARS-CoV-2 mRNA vaccination in 44 patients with MM, who underwent vaccination from 12/17/2020 to 3/18/2021.

**Results:**

Rates adverse reactions were low and consistent with those documented in vaccine trials. Among those on MM therapy, 93% developed detectable anti-receptor binding domain (RBD) antibodies after dose 2, while 94% of patients not on MM therapy seroconverted.

**Conclusions:**

Two-dose SARS-CoV-2 mRNA vaccination is mildly reactogenic and leads to high rates of seroconversion in patients with MM. These findings can provide reassurance to MM patients who are hesitant to receive SARS-CoV-2 mRNA vaccines.

**Supplementary Information:**

The online version contains supplementary material available at 10.1186/s12885-021-09097-5.

## Introduction

Patients with multiple myeloma (MM) have experienced high (34%) inpatient mortality due to COVID-19 [1]. However, patients with MM and other immunocompromised populations were excluded from the SARS-CoV-2 mRNA vaccine trials [2–4]. Lack of information about the safety and immunogenicity of the vaccines in patients with MM may contribute to vaccine hesitancy, and as such these data are critical to patients and their providers.

Reactogenicity of the SARS-CoV-2 mRNA vaccines in immunocompromised populations, such as solid organ transplant (SOT) and rheumatic and musculoskeletal diseases (RMD) populations, appears similar to that of the immunocompetent people studied in the original vaccine trials [2–8]. However, the immunogenicity of the vaccines has been demonstrated to be decreased in these populations [5–8].

Patients with MM are on therapies that dampen the humoral and cellular immune responses, which has been linked to a diminished response to vaccines [9]. One recent study demonstrated 56% seroconversion at least 21 days following the first dose (D1) of the SARS-CoV-2 mRNA vaccine in the MM population, which is substantially lower than the 100% seroconversion observed in the original trials [2–4, 10]. Two other groups found reduced post-D1 neutralizing antibody production in the elderly MM population, relative to healthy controls (20.6% MM vs. 32.5% control after 3 weeks; 78.6% MM vs. 100% control after 5 weeks) [11, 12]. In contrast to these three single-center reports on the mRNA vaccine in patients with MM, the present study features a younger, national sample, as well as the safety and antibody response to two doses of mRNA vaccine [10–12]. We studied the safety and antibody response to two-dose SARS-CoV-2 mRNA vaccination in patients with MM.

## Materials and methods

Patients who reported a diagnosis of MM ≥18 years old without a history of COVID-19 were recruited to participate in this prospective cohort between 12/17/2020–3/18/2021. Recruitment was conducted via a social media campaign. Diagnosis, demographics, and therapeutic regimens were collected via participant report and managed using the REDCap electronic data capture tool, a secure, web-based software platform designed to support data capture for research studies. One week after each dose, participants completed a questionnaire about local (pain, swelling, erythema) and systemic reactions (fatigue, headache, myalgia, chills, fever, diarrhea, vomiting) as well as adverse events (anaphylaxis, incident neurologic diagnoses, and infections including SARS-CoV-2).

One month after dose 2 (D2), participants underwent SARS-CoV-2 antibody testing via the Roche Elecsys® anti-SARS-CoV-2 S enzyme immunoassay which measures total antibody (IgM, IgG) to the SARS-CoV-2 S-receptor binding domain (RBD) protein, the target of the mRNA vaccines. The assay’s detection limits ranged from < 0.4 to > 250 U/mL, with a positive result at > 0.79 U/mL. This study was approved by the Johns Hopkins Institutional Review Board (IRB00248540) and participants provided informed consent electronically.

## Results

We studied 44 patients with MM who received two-dose SARS-CoV-2 mRNA vaccination (Table 1). The median (IQR) age was 64 (57–69) years, 68% were female, 98% were white, and 50% received the Pfizer/BioNTech vaccine while 50% received Moderna. The most common therapeutic regimens included lenalidomide (39%), daratumumab (16%), and pomalidomide (9%), while 17 (39%) were not on therapy.

**Table 1:** Demographic and clinical characteristics of 44 patients with multiple myeloma, stratified by anti-SARS-CoV-2 RBD antibody response to two-dose SARS-CoV-2 mRNA vaccination

	Overall (*n* = 44)	Detectable antibody^**a**^ (*n* = 41)	Undetectable antibody^**a**^ (*n* = 3)
**Age**, median (IQR)	64 (57, 69)	64 (57, 69)	58 (55, 58)
**Female**, no. (%)	30 (68)	28 (93)	2 (7)
**Non-white**, no. (%)	1 (2)	1 (100)	0 (0)
**Vaccine manufacturer**, no. (%)			
Pfizer/BioNTech	22 (50)	21 (95)	1 (5)
Moderna	22 (50)	20 (91)	2 (9)
**Days from vaccine to antibody testing**, median (IQR)	29 (28, 32)	29 (27, 31)	32 (28, 38)
**Therapy**, no. (%)			
Not on therapy	17 (39)	16 (94)	1 (6)
On therapy^b^	27 (61)	25 (93)	2 (7)
Bortezomib	1 (2)	1 (100)	0 (0)
Carfilzomib	1 (2)	1 (100)	0 (0)
Daratumumab	7 (16)	7 (100)	0 (0)
Ixazomib	2 (5)	1 (50)	1 (50)
Lenalidomide	17 (39)	16 (94)	1 (6)
Pomalidomide	4 (9)	4 (100)	0 (0)
Teclistamab	1 (2)	0 (0)	1 (100)

^a^ The percentages in these columns are shown as percent of each category in the overall column. Detectable antibody is defined as an anti-SARS-CoV-2 RBD antibody titer > 0.79 U/mL by the manufacturer

^b^ Since participants could report more than one therapy, the sum of the therapies is greater than the total N

The majority of local and systemic reactions were mild (80% of all local reactions and 71% of all systemic reactions) (Supplemental Fig. 1). The most common local reaction was pain (75% after D1 and 73% after D2). The most common systemic reactions were fatigue (39% after D1 and 64% after D2), headache (32% after D1 and 50% after D2), and myalgia (32% after D1 and 41% after D2). Since vaccination, no participant developed anaphylaxis, incident SARS-CoV-2, or a new neurologic condition or infection.

**Fig. 1:**
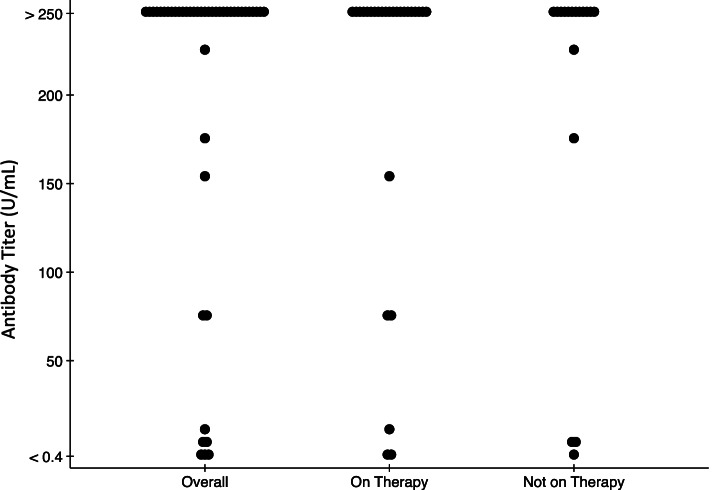
Anti-SARS-CoV-2 RBD antibody titers of 44 patients with multiple myeloma one month after two-dose SARS-CoV-2 mRNA vaccination. Results range from <0.4 to >250 U/mL with detectable antibody defined as an anti-SARS-CoV-2 RBD antibody titer >0.79 U/mL by the manufacturer

Forty-four participants underwent antibody testing at a median (IQR) of 29 (28–32) days after D2 (Table 1, Fig. 1, Supplemental Table 1). Anti-RBD was detectable in 41/44 (93%; 95% CI, 81–99%) overall, 25/27 (93%; 95% CI, 76–99%) of those on therapy, and 16/17 (94%; 95% CI, 71–99%) of those not on therapy. The median titer was above the upper limit of the assay (> 250 U/mL) overall, for those on therapy, and for those not on therapy. The two participants on therapy that did not seroconvert were on teclistamab (a B-cell maturation antigen [BCMA]-CD3 bi-specific antibody in development) and lenalidomide/ixazomib.

## Discussion

In this study of patients with MM, two-dose SARS-CoV-2 mRNA vaccination resulted in expected systemic reactogenicity and high immunogenicity. Local and systemic adverse reactions were mostly mild (80% of local and 71% of systemic reactions), which is a trend consistent with what was reported in the vaccine clinical trials [3, 4]. Local reactions were more common post-D1, and systemic reactions were more reported by more participants post-D2, which is also consistent with what was reported in the vaccine clinical trials [3, 4]. We found high rates of seroconversion after two doses of the vaccine, and these did not vary based on whether patients were on MM therapy (93% seroconversion on therapy vs. 94% seroconversion without therapy). This contrasts with what was observed in another study post-D1, where 74% of patients without therapy seroconverted post-D1, while only 48% of those on therapy seroconverted [11].

While high immunogenicity in MM patients is promising, clinical trials in the general population reported a 100% seroconversion rate for both vaccine types [3, 4]. Median antibody titers in immunocompetent populations in response to the vaccine measured by the same assay used in this study have been found to be > 250 U/mL [13, 14]. Of note, the only participant on teclistamab did not develop an antibody response. Earlier studies found that lack of SARS-CoV-2 seroconversion occurs in patients on B-cell disruptive therapy [15]. Teclistamab induces T cell-mediated destruction of B-cell maturation antigen (BCMA) positive B-cells [16]. Previous work has shown that lenalidomide can boost vaccine effectiveness in MM patients [17]. In this study, 94% (16/17) of the patients on lenalidomide experienced positive seroconversion.

This study is limited by a small size, which limits the ability to compare titers between participants on different regimens. Furthermore, the assay’s low dynamic range makes quantitative comparison of antibody titers difficult. Additionally, monoclonal protein concentrations were not measured, which have been correlated with vaccine response to other vaccines [18]. Due to these limitations, we do not yet know the degree to which detectable antibody levels impact the clinical presentation and severity of COVID-19 in patients with MM. The present study presents evidence that vaccination against SARS-CoV-2 is safe for patients with MM. Additionally, increased anti-RBD antibody levels suggest that vaccination may decrease COVID-19 morbidity and mortality in this population. Social media recruitment and convenience sampling may have influenced the homogeneity of the study, as other papers from our group, with the same recruitment strategy, yielded similar population demographics [6–8]. Future investigations should include larger samples, examine the role of specific medications in vaccine response, and examine cellular immunity.

## Conclusion

In conclusion, while patients with MM are at increased risk of COVID-19 disease burden and need to remain vigilant to protect themselves, this study provides evidence that vaccination with the BNT162b2 and mRNA-1273 vaccines is safe and immunogenic in patients with MM. These results support the recommendation of SARS-CoV-2 mRNA vaccination for patients with MM.

## Supplementary Information

**Additional file 1:** **Supplemental Figure 1.** Local site and systemic adverse reactions in 44 patients with multiple myeloma within 7 days of dose 1 and dose 2 of the SARS-CoV-2 mRNA vaccine. **Supplemental Table 1.** Anti-SARS-CoV-2 RBD antibody titer of 44 patients multiple myeloma 1 month after two-dose SARS-CoV-2 mRNA vaccination by demographics and clinical characteristics.

## Data Availability

The datasets used and/or analysed during the current study available from the corresponding author on reasonable request.

## Abbreviations

MM: Multiple myeloma
SOTR: Solid organ transplant recipients
D1: Dose 1
D2: Dose 2
