# Supplementary material for: Safety and antibody response to two-dose SARS-CoV-2 messenger RNA vaccination in patients with multiple myeloma
Source: BMC Cancer. 2021 Dec 27;21:1354. doi: 10.1186/s12885-021-09097-5 (PMC8711688; doi:10.1186/s12885-021-09097-5)
Supplement: Supplementary file 1 — Additional file 1: Supplemental Figure 1. Local site and systemic adverse reactions in 44 patients with multiple myeloma within 7 days of dose 1 and dose 2 of the SARS-CoV-2 mRNA vaccine. Supplemental Table 1. Anti-SARS-CoV-2 RBD antibody titer of 44 patients multiple myeloma 1 month after two-dose SARS-CoV-2 mRNA vaccination by demographics and clinical characteristics. [file 12885_2021_9097_MOESM1_ESM.pptx]

## Slide 1
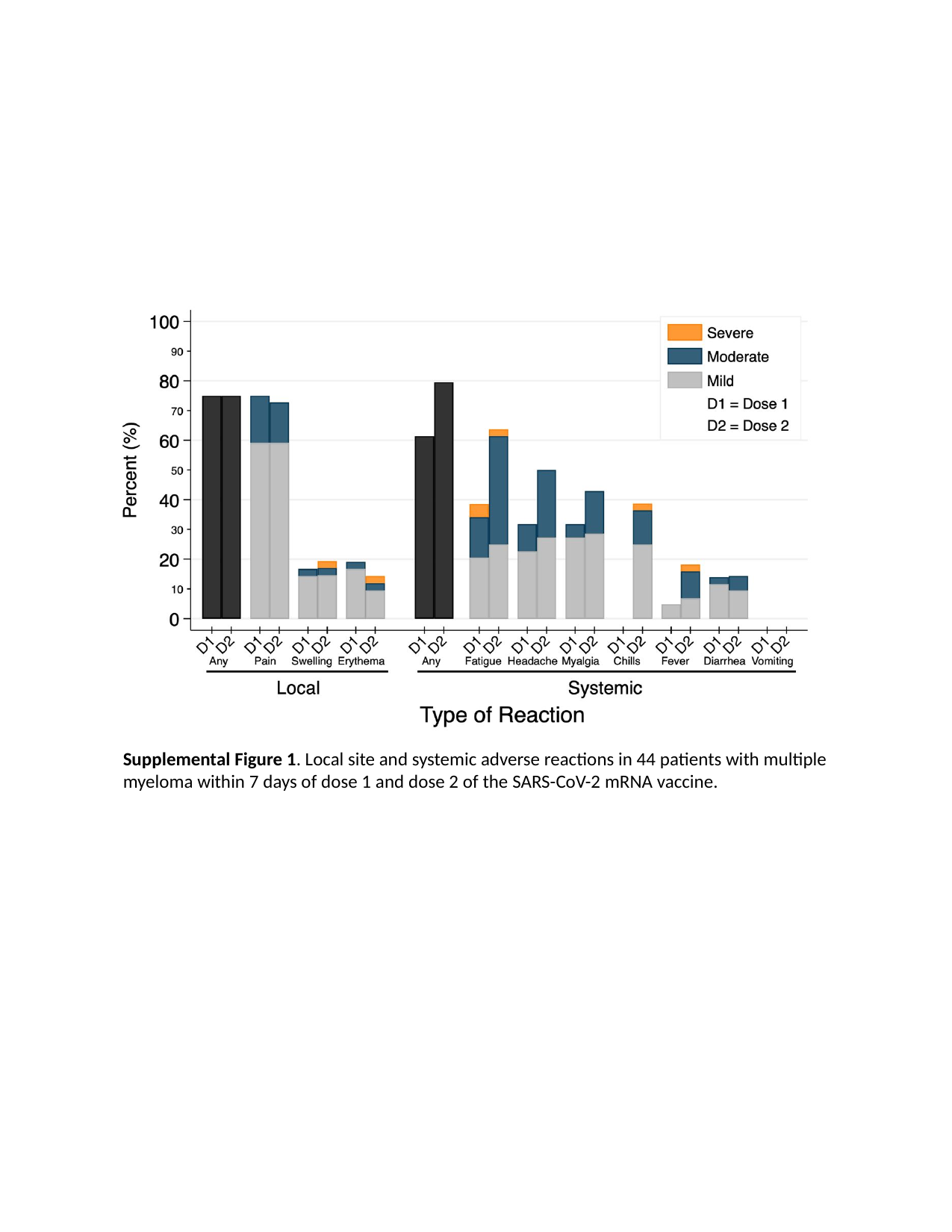

Supplemental Figure 1. Local site and systemic adverse reactions in 44 patients with multiple myeloma within 7 days of dose 1 and dose 2 of the SARS-CoV-2 mRNA vaccine.

## Slide 2
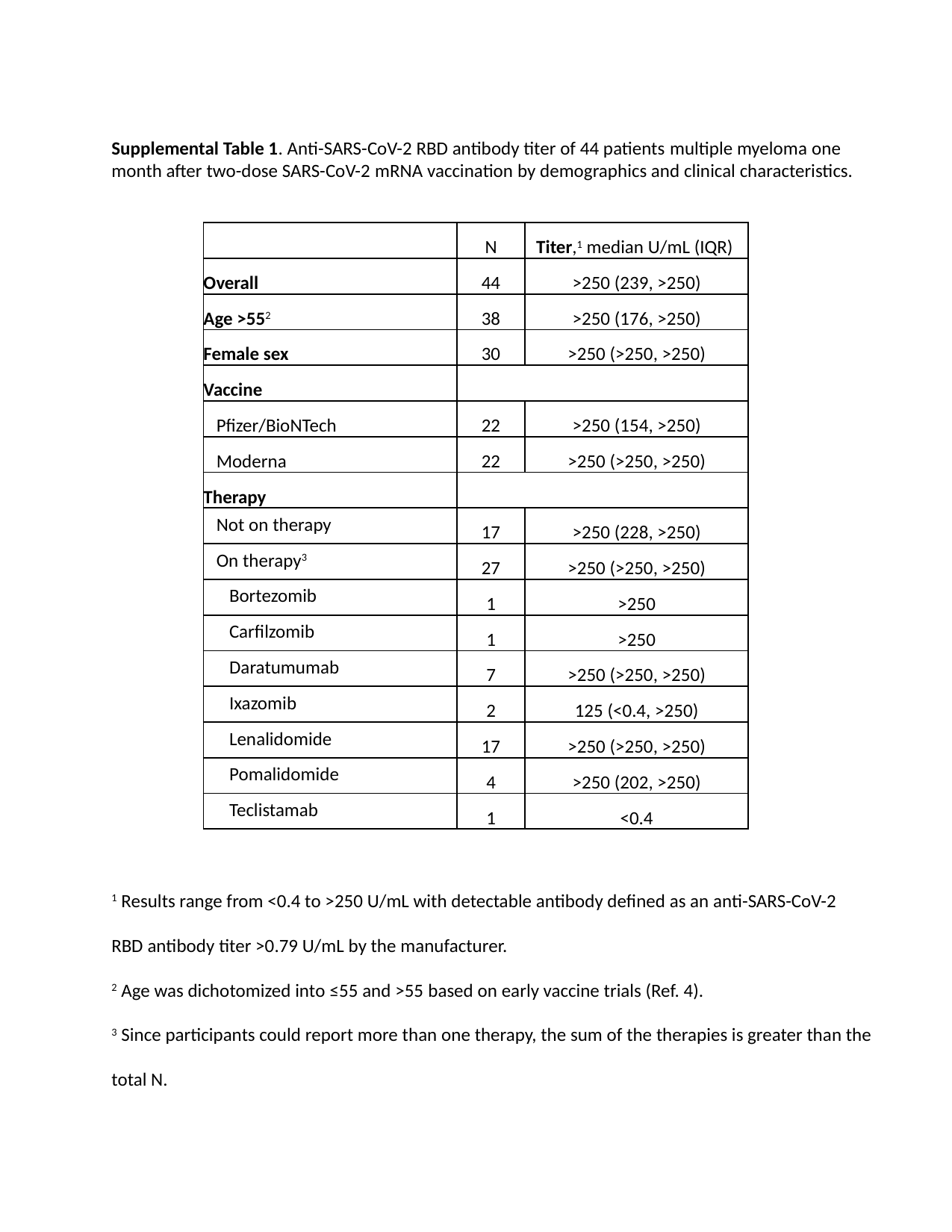

Supplemental Table 1. Anti-SARS-CoV-2 RBD antibody titer of 44 patients multiple myeloma one month after two-dose SARS-CoV-2 mRNA vaccination by demographics and clinical characteristics.
| | N | Titer,1 median U/mL (IQR) |
| --- | --- | --- |
| Overall | 44 | >250 (239, >250) |
| Age >552 | 38 | >250 (176, >250) |
| Female sex | 30 | >250 (>250, >250) |
| Vaccine | | |
| Pfizer/BioNTech | 22 | >250 (154, >250) |
| Moderna | 22 | >250 (>250, >250) |
| Therapy | | |
| Not on therapy | 17 | >250 (228, >250) |
| On therapy3 | 27 | >250 (>250, >250) |
| Bortezomib | 1 | >250 |
| Carfilzomib | 1 | >250 |
| Daratumumab | 7 | >250 (>250, >250) |
| Ixazomib | 2 | 125 (<0.4, >250) |
| Lenalidomide | 17 | >250 (>250, >250) |
| Pomalidomide | 4 | >250 (202, >250) |
| Teclistamab | 1 | <0.4 |
1 Results range from <0.4 to >250 U/mL with detectable antibody defined as an anti-SARS-CoV-2 RBD antibody titer >0.79 U/mL by the manufacturer.
2 Age was dichotomized into ≤55 and >55 based on early vaccine trials (Ref. 4).
3 Since participants could report more than one therapy, the sum of the therapies is greater than the total N.
